# Supplementary material for: Tonic and early interferons defend against respiratory viruses in primary human lung organoid-derived air-liquid interface cultures
Source: J Virol. 2026 May 20;100(6):e02104-25. doi: 10.1128/jvi.02104-25 (PMC13288630; doi:10.1128/jvi.02104-25)
Supplement: Supplemental material — Fig. S1 to S5; legends for Videos S1 to S4. [file jvi.02104-25-s0006.docx]

**Supplementary Information**

**Tonic and early interferons defend against respiratory viruses in primary human organoid-derived bronchial epithelial air-liquid interface cultures**

Rinu Sivarajan, Paul C Kirchgatterer, Jan Lawrenz, Eszter Tanner-Matiz, Jessica Lindenmayer, Verena Renz, Tapan Joshi, Heike Oberwinkler, Thorsten Walles, Giorgio Fois, Alexander Kleger, Manfred Frick, Jan Münch, Moritz M Gaidt, Maria Steinke, Konstantin MJ Sparrer

**Figures S1-5.**

**Supplementary Video Legends.**


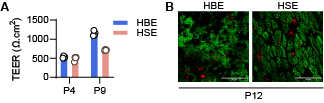


**Figure S1.** (**A**) Barrier integrity comparison of HBE and HSE from lung organoids across passages. TEER measurements of HBE and HSE derived from lung organoids at passages 4 (P4) and 9 (P9) over serial passaging. Bars represent the mean±SEM. n=3 (independent experiments). (**B**) Representative immunofluorescence images of HBE and HSE generated from passage 12 organoids stained for ciliated (acTub+, green) and secretory (MUC5B+, red) cells. Scale bar, 50 µm.


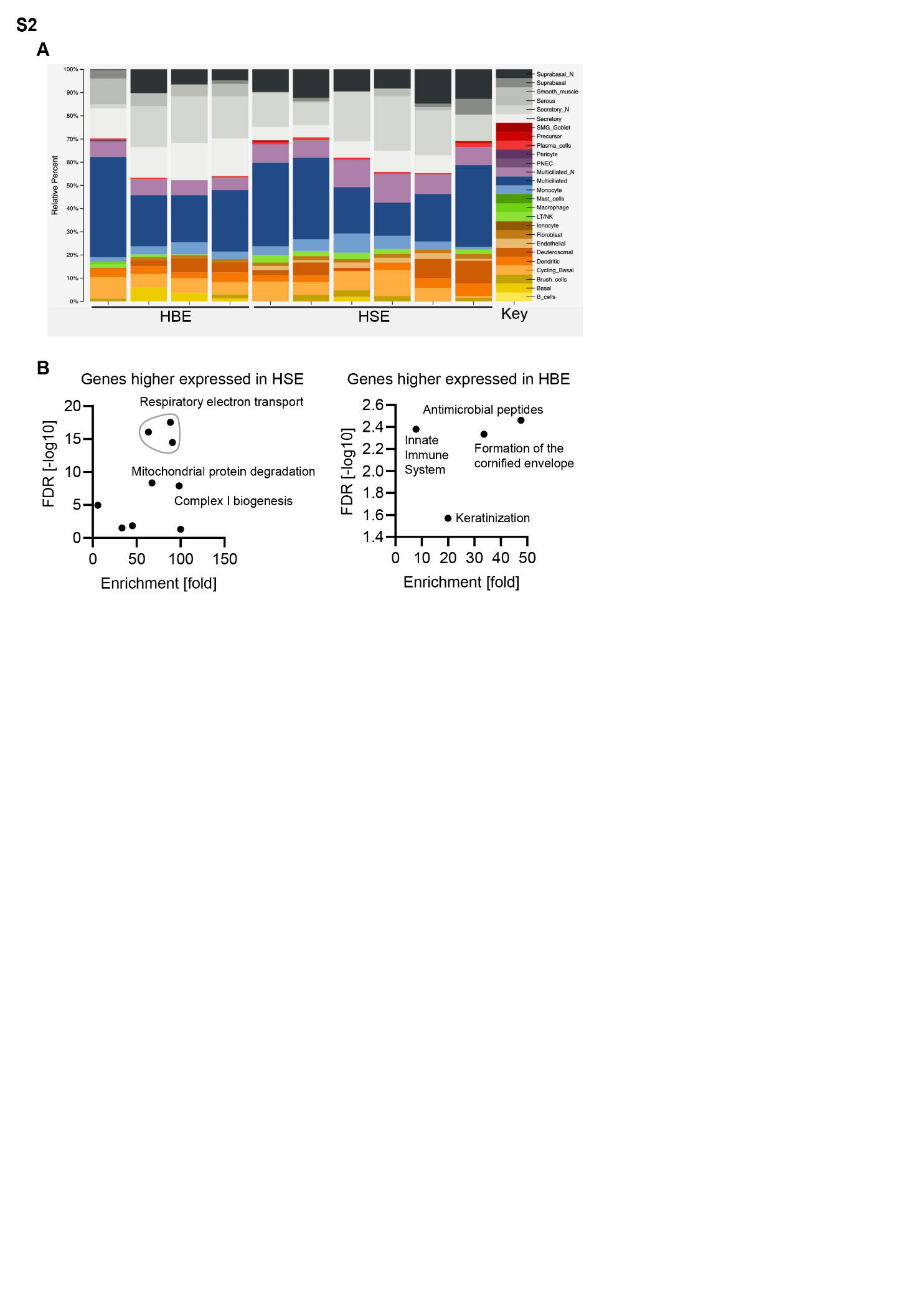


**Figure S2.** (A) CIBERSORTx output of transcriptomic comparison of cell types present in bronchial and small airway epithelium matched to the healthy lung atlas. Data are from n=3 independent experiments (B) Gene Ontology analysis of the Top 40 genes by variance in Fig. 3D. PANTHER Overrepresentation Test.

**
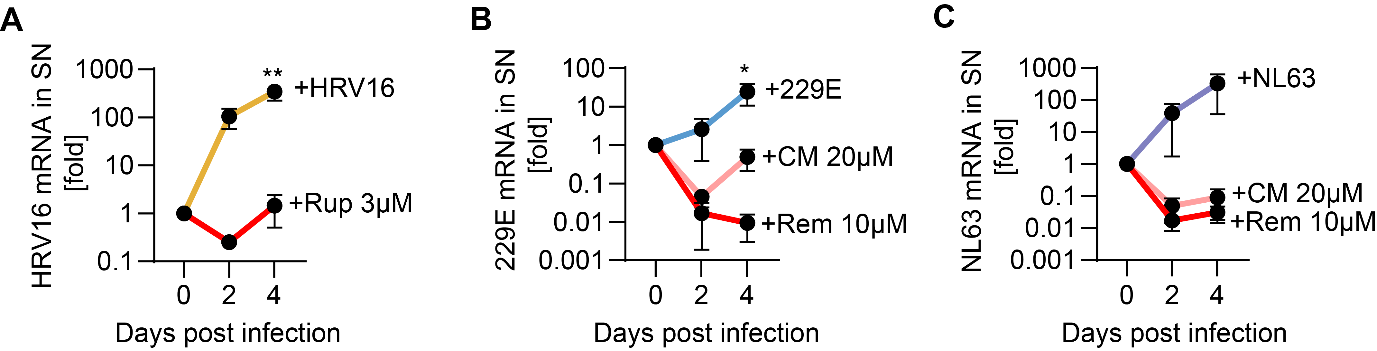
 Figure S3.** Human bronchial epithelial models as an in vitro model for testing anti-viral drugs (A-C). The apical side of differentiated HBEs was exposed to viral inoculum (MOI 0.01) for 3 h before removal and apical washing. Respective inhibitor was added in parallel to the basal compartment (10 µM Remdesivir, 20 µM Camostat mesylate, 3 µM Rupintrivir) and replenished with medium changes at 2- and 4 dpi. At the indicated days post-infection, apical washes were collected and subjected to RT-qPCR. The data are from the mean ±SEM of 3 independent experiments. Statistical analysis was performed using two-way ANOVA with Tukey’s multiple comparisons test. **p = 0.0067; *p = 0.0400.


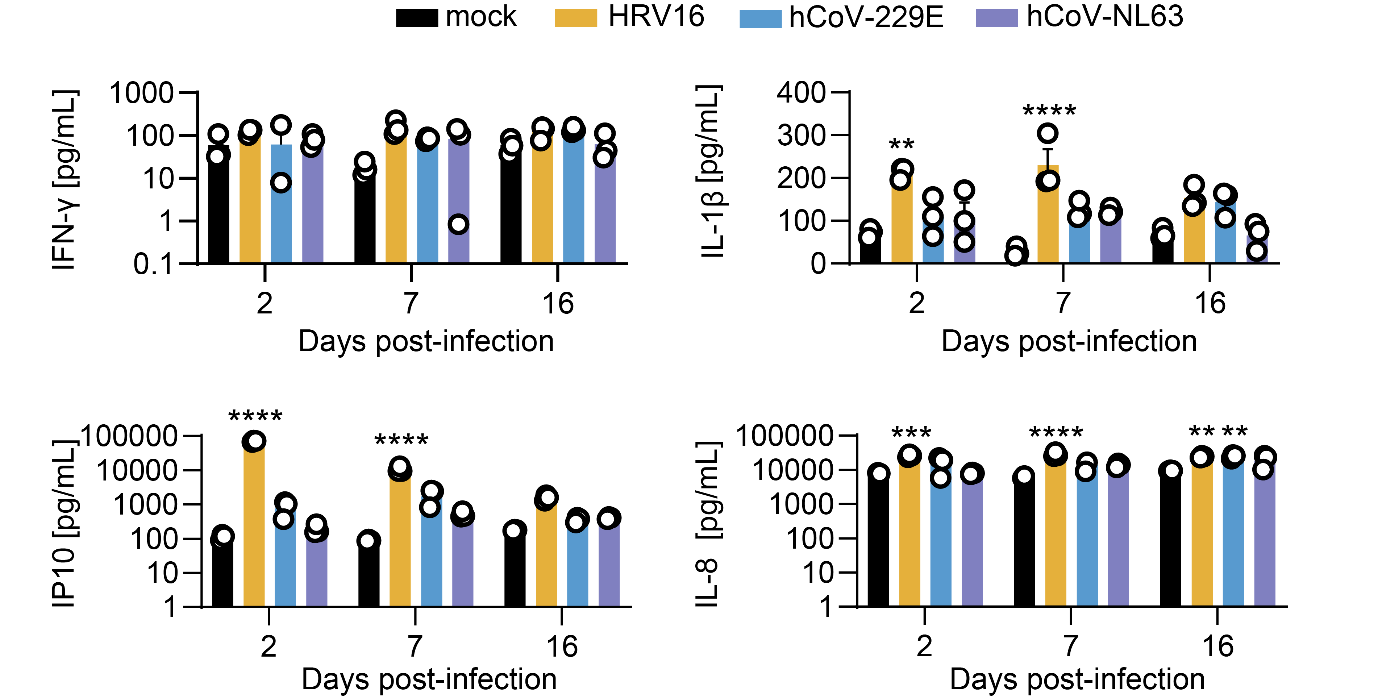


**Figure S4.** Temporal cytokine dynamics during early and late infection phases. Cytokine levels from the basal compartment of human bronchial epithelial models infected with HRV16, 229E, or NL63 measured at 2, 7 and 16 dpi. Bars represent the mean±SEM. n=3 (independent experiments). Statistical analysis was performed using two-way ANOVA with Tukey’s multiple comparisons test. * indicates significance (****p< 0.0001, *** p= 0.0003,** p= 0.008.


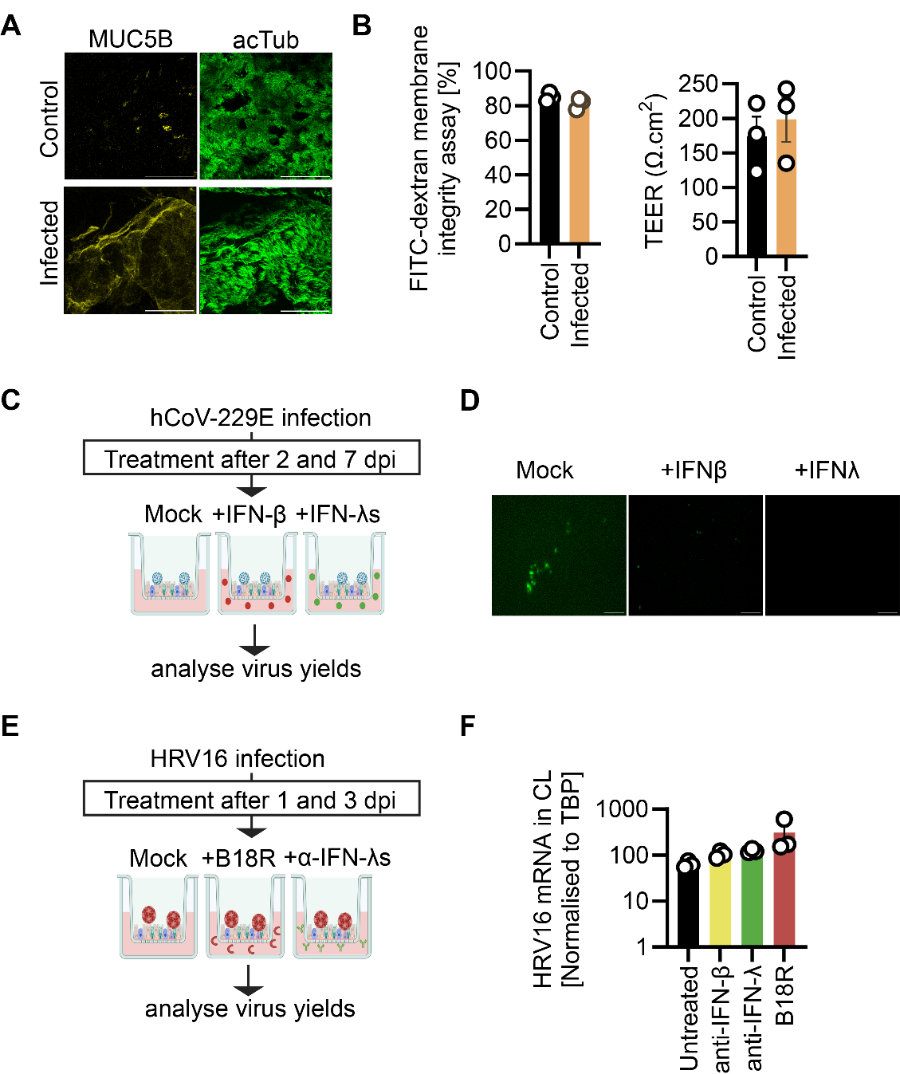


**Figure S5.** Long-term monitoring of infection-associated changes in the human bronchial epithelium. (A) Confocal microscopy of bronchial epithelial cultures after infection clearance stained for secretory cells (MUC5B+), and an intact ciliated cell layer (acTub⁺). Scale bar, 100 µm. (**B**) FITC-dextran permeability assay and TEER measurements demonstrated comparing membrane integrity between control and infection-cleared cultures after 35 dpi. The data represent the mean of n=3 ±SEM (3 donors). (**C**) Schematic representation of exogenous IFN addition in HCoV-229E-GFP infected HBEs at 2 and 7 dpi post-infection (MOI 0.01). (**D**) Representative fluorescent images of HBEs infected with HCoV-229E-GFP at 7 dpi. Scale bar, 100 µm. (**E**) Schematic representation of IFN depletion in HBEs using neutralising antibodies or B18R after 1 and 3 dpi with HRV16 (MOI 0.01). (**F**) HRV16 RNA content in IFN-depleted cultures. RT-qPCR of cell lysates from bronchial epithelial cultures infected with HRV16 after 1 dpi analysed for viral RNA in tonic IFN-depleted groups compared to untreated controls. HRV16 gene expression was normalised to the housekeeping gene TBP. Bars represent the mean±SEM. n=3 (independent experiments).

**Supplemental Video 1.** Functional ciliary activity in HBE cultures. Synchronised movement of Dynabeads Protein G particles across the epithelial surface, indicating a fully differentiated, healthy status of the cultures, recorded using high-speed video microscopy.

**Supplemental Videos 2–4.** Representative live-cell imaging of HBE cultures at 16 dpi with respective viruses at a MOI of 0.01 shows no large-scale cell death or a visible cessation of ciliary beating. Supplementary video 2 represents HRV16-infected HBE; Supplementary video 3 represents 229E-infected HBE; Supplementary video 4 shows HBE infected with NL63.
